# Isolation and Characterization of a Newly Discovered Phage, V-YDF132, for Lysing *Vibrio* *harveyi*

**DOI:** 10.3390/v14081802

**Published:** 2022-08-17

**Authors:** Shaozhu Kang, Luhao Zhang, Jiaming Liao, Dongzhuo Zhang, Siting Wu, Xin Zhang, Qiwei Qin, Jingguang Wei

**Affiliations:** 1 College of Marine Sciences, South China Agricultural University, Guangdong Laboratory for Lingnan Modern Agriculture, Guangzhou 510642, China; 2 Guangdong Winsun Biological Pharmaceutical Co., Ltd., Guangzhou 511356, China; 3 Laboratory for Marine Biology and Biotechnology, Pilot National Laboratory for Marine Science and Technology (Qingdao), Qingdao 266000, China; 4 Southern Marine Science and Engineering Guangdong Laboratory (Zhuhai), Zhuhai 528478, China; 5 Department of Biological Sciences, National University of Singapore, Singapore 117543, Singapore

**Keywords:** *Vibrio harveyi*, *Vibrio* phage, lytic bacteriophage, aquaculture, V-YDF132

## Abstract

A newly discovered lytic bacteriophage, V-YDF132, which efficiently infects the pathogenic strain of *Vibrio harveyi*, was isolated from aquaculture water collected in Yangjiang, China. Electron microscopy studies revealed that V-YDF132 belonged to the *Siphoviridae* family, with an icosahedral head and a long noncontractile tail. The phage has a latent period of 25 min and a burst size of 298 pfu/infected bacterium. V-YDF132 was stable from 37 to 50 °C. It has a wide range of stability (pH 5–11) and can resist adverse external environments. In addition, in vitro the phage V-YDF132 has a strong lytic effect on the host. Genome sequencing results revealed that V-YDF132 has a DNA genome of 84,375 bp with a GC content of 46.97%. In total, 115 putative open reading frames (ORFs) were predicted in the phage V-YDF132 genome. Meanwhile, the phage genome does not contain any known bacterial virulence genes or antimicrobial resistance genes. Comparison of the genomic features of the phage V-YDF132 and phylogenetic analysis revealed that V-YDF132 is a newly discovered *Vibrio* phage. Multiple genome comparisons and comparative genomics showed that V-YDF132 is in the same genus as *Vibrio* phages vB_VpS_PG28 (MT735630.2) and VH2_2019 (MN794238.1). Overall, the results indicate that V-YDF132 is potentially applicable for biological control of vibriosis.

## 1. Introduction

*Vibriosis* is one of the widespread and devastating diseases of marine and captive fish, and it causes high mortality and significant economic losses [1]. Naturally occurring in marine habitats, *Vibrio harveyi* has become an important pathogen for wild and farmed marine fish and invertebrates [2,3], particularly in warm waters of Asia, southern Europe, and Europe. Traditionally, antibiotics have been commonly used for the prophylaxis and treatment of *V*. *harveyi* infection in aquaculture. Moreover, several cases of antibiotic-resistant strains of *V*. *harveyi* have been reported in recent years [4,5,6,7,8], along with alerts from the World Health Organization (WHO) regarding the development of antibiotic resistance in marine pathogens [9]. This emergence of antimicrobial resistance in pathogens affecting aquaculture has prompted a search for alternative therapeutic approaches. Although vaccination is an option to control infectious diseases, very few vaccines have been authorized for use in aquaculture. This approach is also not feasible for crustaceans and mollusks and has low or no ecological effect on juvenile fish [10]. This makes it imperative to evaluate new approaches or complementary alternatives. In this context, bacteriophages (or phages) have been considered potential therapeutic agents for treating pathogenic *Vibrio* infections in aquaculture [11,12,13,14,15,16]. The mechanisms by which phages destroy bacterial cells differ greatly from those used by antibiotics [17]. Therefore, there is an urgent need to isolate new and specific phages to combat bacterial pathogens.

Bacteriophages are the most abundant biological form in the biosphere, with a total abundance of approximately 10^31^ [18]. They consist of DNA or RNA wrapped in a protein coat [19]. The phage genome contains many novel gene sequences of unknown function and may represent the most extensive new gene pool [20]. Phages are viruses that specifically infect bacteria; they are highly host-specific and are environmentally friendly alternatives to antibiotics that control and kill pathogenic bacteria. Over the past 20 years, numerous in vitro trials have been conducted to determine the effects of phage therapy on pathogenic bacteria in fish. Several in vivo studies have also evaluated the potential of phages to control bacterial infections in aquaculture, including the use of phages to effectively combat multidrug-resistant bacteria [21,22]. Recent studies have reported the use of phages to control many diseases caused by different species of *Vibrio*. In particular, treatment of *Penaeus monodon* larvae suffering from luminescent vibriosis caused by *V. harveyi* with phages from the *Siphoviridae* family (the bacteriophages were added to the tank water) resulted in 85% survival of the larvae, as compared to 65–68% of animals surviving after antibiotic treatment [23]. Another example is a lytic phage (also added to tank water) that was successfully used to treat Atlantic salmon (*Salmo salar*) infected with *V. anguillarum*; the treatment resulted in a survival rate of up to 100%, while less than 10% of the untreated fish survived the disease [24]. A noteworthy finding is that the addition of a cocktail of phages belonging to the *Siphoviridae* family to water effectively controlled vibriosis caused by *Vibrio* sp. The phage Va-F3 increased the survival of the shrimp *Litopenaeus vannamei* from 20% (untreated group) to 91.4% (phage-treated group); the success rate of the phage treatment was higher than that obtained with antibiotic therapy [25]. Thus, phage therapy is considered an environmentally friendly approach with a high potential to reduce and control drug-resistant *V*. *harveyi* in aquaculture.

The present study reports the biological and genomic characterization of a lytic siphovirus, named V-YDF132. The phage was isolated from aquaculture water collected in Yangjiang, China, and its host is a pathogenic *V. harveyi* strain which was isolated from diseased grouper. To elucidate the novelty of this phage, phylogenetic analysis based on the major capsid protein and the terminase large subunit, genomic analysis, and comparative in silico proteomic analysis of V-YDF132 and other related phages were performed.

## 2. Materials and Methods

### 2.1. Bacterial Strains, Phage Isolation, and Purification

Bacterial strains used in the present study were isolated from samples of diseased grouper in aquafarm (Yangjiang, Guangdong province, China). The *Vibrio* phages were isolated from the aquaculture water collected from the same aquafarm. The water sample was sterilized by passing through a 0.22 μm syringe filter. The isolation step was performed as described previously [26,27]. To enrich the target phage in the water sample, 20 mL of the filtered water sample and 20 mL of 3-fold Luria–Bertani (LB) medium were mixed with 1 mL of host bacterial broth and placed on a shaking incubator at 37 °C for 24 h. The mixture was centrifuged at 8000× *g* for 10 min, and the supernatant was passed through a 0.22 μm syringe filter to remove the residual bacterial cells; the process was repeated at least three times. The bilayer plate method was used to isolate and detect the phage. Briefly, 100 μL of the phage enrichment solution was diluted in a 10-fold gradient by using SM buffer or 2216E liquid medium as the diluent, mixed with 100 μL of the host bacterial solution, and incubated at 37 °C for 15 min (to allow the phage to adsorb entirely on the bacterial cells). Approximately 5 mL of the top agar layer (LB with 0.65% agar) at 45 °C was then added, and the mixture was poured onto an LB plate. The plates of the phage V-YDF132 were then incubated at 37 °C for 5 or 8 h to obtain phage plaques. Then, the largest and most translucent plaque was cored, plaques were purified three times and resuspended in 1 mL of sterile SM buffer. Purified phages were stored in SM buffer at 4 °C for several months for further processing.

### 2.2. Phage Titer Assay

The purified phage was diluted 10 times to a suitable gradient for double-layer plate detection, and the plates inoculated with the phage were incubated at 37 °C for 6–8 h until phage spots appeared; the number of phage spots was then recorded. The assay was performed with three replicate plates for each dilution, and suitable dilutions were made to obtain 30–300 phage spots on each plate. Lastly, we calculated the titer of the original phage culture by using the following formula: number of plaques × 10 × reciprocal of counted dilution = pfu/mL.

### 2.3. CsCl Gradient Purification and Electron Microscopic Analysis

CsCl density gradient centrifugation of the phage concentrates was performed according to a previous method with some modifications [28]. A high-titer phage lysate was precipitated using polyethylene glycol (10% *w*/*v* PEG8000, 0.5 M NaCl) at 4 °C overnight and centrifuged at 12,000× *g* for 20 min at 4 °C. The pellet was then resuspended in SM buffer. The resulting phage preparation was placed on a CsCl step gradient comprising 1.3, 1.5, and 1.7 g/mL density layers and spun on an SW 32 Ti rotor (Optima XE-100 ultracentrifuge, Beckman Coulter, Brea, CA, USA) at 174,900× *g* (34,200 rpm) for 3 h at 4 °C. Phage pellets located near the density band of 1.5 g/mL were collected. Subsequently, 10 μL of purified phage (>10^10^ pfu/mL) was stained with 2% uranyl acetate. After drying at room temperature, the grids were examined using a transmission electron microscope at an acceleration voltage of 120 kV with a CCD camera.

### 2.4. Phage Host Range Determination

The host range of V-YDF132 was determined by the spot assay and verified by the double layer agar method. The test strains listed in Table 1 were individually cultured to an OD600 of about 0.6, an aliquot of 100 μL of each culture and 5 mL of LB containing 0.65% agar was mixed and poured onto an LB agar plate. After standing for 5 min at room temperature, 10 μL of the 10-fold serially diluted phages in LB were spotted onto LB soft agar. The plates were then incubated at 37 °C and examined for plaques after 8–18 h of incubation. Bacterial sensitivity to phage was confirmed by a clear lysis zone at the spot; based on the clarity of the lysis zone, the bacterial strains were divided into two groups: clear lysis zone (+) and no lysis zone (−).

### 2.5. Determination of Optimal Multiplicity of Infection and One-Step Growth Curve of the Phage

The multiplicity of infection is the ratio of the number of phages and bacteria at the time of infection. Based on the proportion of infection, ratios of 0.0001, 0.001, 0.01, 0.1, 1, 10, and 100, plus 50 μL of phage and 50 μL of *V. harveyi* cells (10^8^ CFU/mL) were added to 900 μL of LB. The mixtures were then shaken at 37 °C for 4–6 h, centrifuged at 12,000 rpm for 3 min at room temperature for cell removal, and passed through a 0.22 μm membrane filter to remove the supernatant. The phage titer was determined by a soft agar overlay method [29]. The dilution with the highest phage titer was the optimal multiplicity of infection (MOI).

The phage solution and the host bacterial suspension were mixed in the ratio of optimal MOI. After phage adsorption for 10 min at 37 °C, the mixtures were centrifuged at 12,000 rpm for 10 min to remove any unadsorbed phage particles before resuspending the cell pellets in 10 mL of 2216E liquid medium. Immediately, the sample was shaken and incubated at 37 °C, and aliquots were collected at different time intervals over 80 min. These aliquots were immediately diluted, and phage titers were determined using the double-layer agar method. Three independent experiments were performed for each assay. The assays revealed latent periods and burst size. Latent periods are defined as the time interval between the adsorption and the beginning of the first burst. Additionally, the burst size is calculated as the ratio of the final number of released phage particles to the initial number of infected bacterial cells.

### 2.6. Phage Heat and pH Stability Test

The effects of environmental factors, including pH and temperature, on the phage, were tested according to the methods described by Kim et al. [30], with slight modifications. For the thermal stability test, 500 μL of phage solution was incubated statically at various temperatures (37, 40, 50, 60, 70, and 80 °C) for 20, 40, and 60 min. After the completion of the incubation period, the solution was immediately removed and cooled on an ice bath, and the phage titer was determined by the double-layer plate method; the assay was repeated three times to determine the thermal stability of the phage titer at different temperatures. For the pH stability test, 100 μL filter-sterilized phage samples (10^8^ pfu/mL) were inoculated in 1 mL of 2216E liquid medium (pH 7.5), adjusted to pH 2 to 12 with 1 M NaOH and 1 M HCl, and incubated at 37 °C for 1 h statically. The phage titer was then determined with the double-layer agar technique.

### 2.7. In Vitro Bactericidal Assay

Single bacterial colonies grown overnight on TCBS solid agar plates were selected and inoculated in a 2216E liquid medium, and the medium was incubated at 37 °C with shaking (120 rpm) until the exponential growth phase was achieved. The concentration of the bacterial solution was then measured, and the suspension was diluted to 10^8^ CFU/mL and centrifuged at 4 °C, 8000× *g* for 5 min. The supernatant was discarded, and the cell pellet was resuspended in an equal volume of 2216E medium. The measurement was performed on sterile 96-well plates using a growth curve measuring instrument by monitoring the optical density at 600 nm (OD_600_). Wells were loaded with 100 μL of the resuspension solution and then mixed with 100 μL of phage lysates (MOI 0.1). For this assay, two control samples were included: the bacteria-only control inoculated with *V. harveyi* alone, and the positive control inoculated with bacteria and doxycycline (100 mg/L). The plate was placed in the instrument and incubated at 30 °C with orbital shaking. The growth curves were monitored in real-time over 12 h, and OD_600_ readings were taken every 1 h. Each condition was assessed in triplicate.

### 2.8. DNA Extraction and Genome Sequencing

The genomic DNA of the bacteriophage was extracted with the Virus DNA Kit (OMEGA) by using DNase and RNase. The phage nucleic acids were detected by electrophoresis [31]. Subsequently, libraries with an average length of 350 bp were constructed using the Nextera XT DNA Library Preparation Kit. The libraries were then sequenced on the Illumina Novaseq 6000 platform. The raw sequence data were edited using the NGS QC Tool Kit v2.3.3 [32], and the high-quality data were then assembled into a phage genome by using a de novo assembler SPAdes v3.11.0 [33].

### 2.9. Bioinformatics Analysis

Open reading frames (ORFs) were predicted using GeneMark.hmm [34,35]. First, the nucleotide sequence data of the phages were searched using the BLASTN tool available on the National Center for Biotechnology Information (NCBI) website. Putative transfer RNA (tRNA)-encoding genes were detected using the tRNAscan-SE [36]. Predicted virulence factors and antibiotic genes were examined using searches against the Virulence Factors Database and the Antibiotic Resistance Gene Database, respectively. The BLASTN comparison tool in the pyani 0.2.11 package was used to determine the average nucleotide identity (ANI) among all the paired phage genome combinations, and interactive heat maps were drawn using heatmaply to identify the phages [37]. Next, ORFs were annotated using the BLASTP algorithm against the non-redundant (nr) protein database available on the NCBI website and eggNOG-Mapper [38]. Comparisons of multiple phage genome sequences were visualized using the BLAST Ring Image Generator (BRIG) with default settings [39]. Comparative genomic analysis was performed using the Easyfig 2.2.5 visualization tool. Finally, phylogenetic analysis was performed with MEGA 6.0 based on major capsid proteins and terminator enzyme large subunits by using the neighbor-joining method with 1000 bootstrap replicates [40].

### 2.10. Statistical Analysis

Data are expressed as the mean and standard deviation. Statistical analysis was performed using GraphPad prism software (8.4.3).

### 2.11. Nucleotide Sequence Record Number

The sequence data of the *Vibrio* phage V-YDF132 have been deposited in GenBank under the accession number ON075462.1.

## 3. Results and Discussion

### 3.1. Origin and Morphology of the Phage V-YDF132

**Figure 1:**
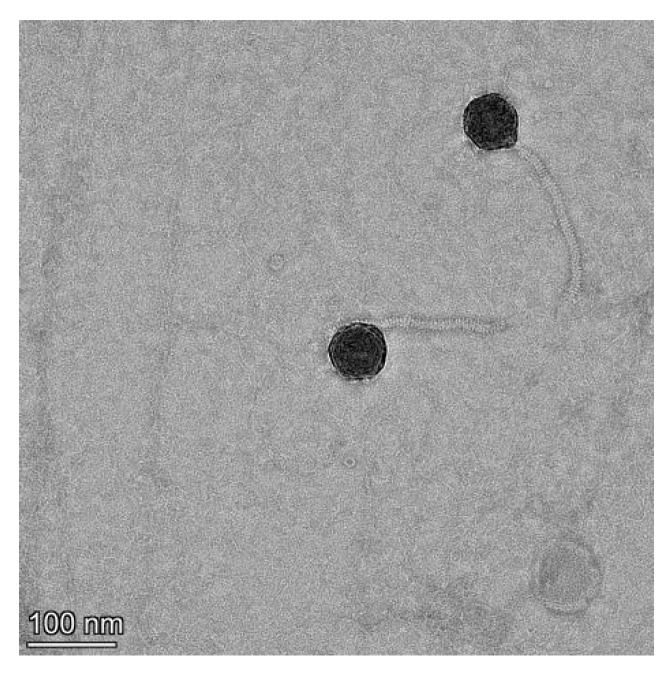
Transmission electron microscopy of V-YDF132. Scale bar represents 100 nm.

A lytic phage strain, named V-YDF132, was isolated from the collected aquaculture water. The phage spot was observed by the double-layer plate method, and the plaque size formed by the phage V-YDF132 was approximately 2 mm in diameter, with a bright and translucent center and a halo around it. The phage morphology observed by transmission electron microscopy revealed that V-YDF132 had an icosahedral head (~69 nm) and a noncontractile sheathed tail (~160 nm) (Figure 1), so it may belong to the *Siphoviridae* family based on the morphology and size of the phage [41].

### 3.2. Host Range of Phage V-YDF132

Among the 16 bacterial strains tested in this study (except for YDF132), the phage V-YDF132 could infect *V. harveyi* YF4-2. The results are shown in Table 1. *V. harveyi* YF4-2 and YDF132 are the same origin, so V-YDF132 may be highly specific. This high specificity may show a potentially unknown recognition mechanism between V-YDF132 and the host strain.

### 3.3. Optimal MOI and One-Step Growth Curve

**Figure 2:**
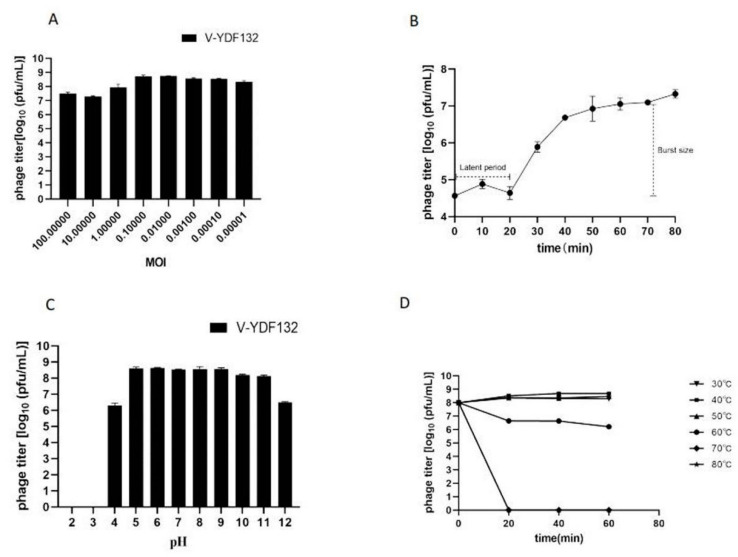
Biological characterization of V-YDF132. (**A**) Multiplicity of infection; (**B**) kinetics of progeny production in a single life cycle; (**C**) pH stability of V-YDF132. The phages were incubated for 1 h at different pH values; (**D**) thermal stability of the phages treated with different temperatures at different time points. Data points represent the mean values and standard deviations (SD) from three independent experiments.

As shown in Figure 2A, the difference between the efficiency of V-YDF132 in infecting hosts at an MOI of 0.1 or 0.01 was minimal, and the efficiency of phage infection of the host remained constant when the MOI was lower than 0.01. Hence, the optimal MOI of V-YDF132 is between 0.1 and 0.01. The one-step growth curve of the phage is a conventional method to describe the growth pattern of virulent phages [42]. The one-step growth curve of the phage V-YDF132 propagated on *V. harveyi* YDF132 was plotted and is shown in Figure 2B, the results showed that the latent period of the phage V-YDF132 was approximately 20 min and a burst size of 298 pfu/cell. Previously, the burst size of the phage P23 was reported to be approximately 24 pfu/cell, with a latent period of 30 min [43]. Another phage, BUCT549, has a latent period of approximately 30–40 min and an average burst size of 141 viral particles per infected cell [44]. The latent period of the Vibrio phage, vB_VcaS_HC, was long at 1.5 h but exhibited a large burst size of 172 pfu per infected cell [45].

### 3.4. Heat and pH Stability

In the present study, we tested the effect of pH and temperature on the stability of the phage V-YDF132 by determining the change in the number of plaque-forming units (pfu). Plaque count showed that the phage V-YDF132 exhibited stability over a wide range of pH (5–11) (Figure 2C). The number of phages decreased by two orders of magnitude when pH decreased to 4.0, and the phages did not show any activity when pH decreased to 3.0. However, the number of the phage V-YDF132 decreased by only two orders of magnitude when pH increased to 12, which indicates that the phage V-YDF132 was more tolerant to alkaline conditions. The thermal stability test showed that the phage V-YDF132 was stable at 37, 40, and 50 °C for 1 h (Figure 2D). When the temperature reached 60 °C and the incubation time reached 20 min, the number of phages decreased by one order of magnitude, and there was a slight decrease in the number of phages with progression in time. Although V-YDF132 was not resistant to higher temperatures, it still showed better stability than the previously reported phage IME271 (40 °C) [46].

### 3.5. In Vitro Lytic Effect

**Figure 3:**
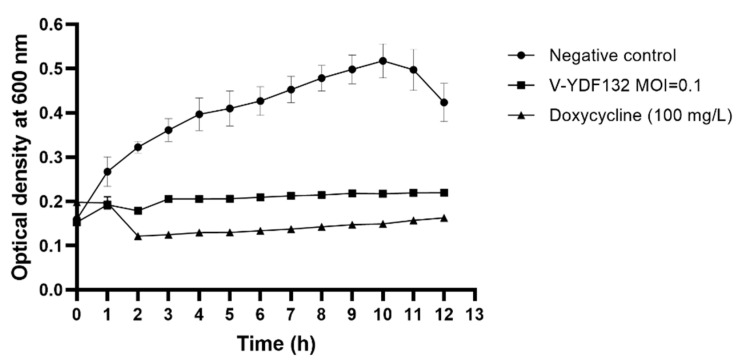
Killing curves of a *Vibrio harveyi* strain by phage V-YDF132 at the optimal MOI (MOI = 0.1). The values represent mean of three determinations.

The in vitro lytic effect of V-YDF132 on its host bacteria was tested by infecting fresh *V. harveyi* in the exponential growth phase. As shown in Figure 3, the growth curve indicated that V-YDF132 very effectively infected the host, with almost no bacterial growth after infection. Thus, the great bactericidal activity revealed that V-YDF132 is a strong candidate with potential for biological control of *V. harveyi* disease.

### 3.6. Phage Genome Characterization and Phylogenetic Analysis

**Figure 4:**
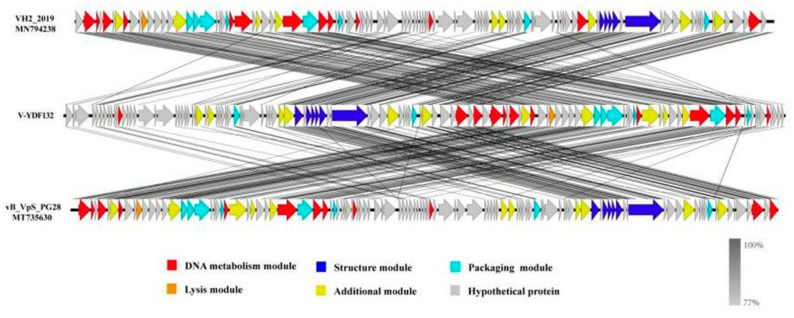
Multiple sequence alignment of phage genomes. The whole genomes of the *Vibrio* phage vB_VpS_PG28 (MT735630.2) and VH2_2019 (MN794238.1) and V-YDF132 were compared at the DNA level by using Easyfig. The black shading indicates sequence similarities between the genomes. The predicted functions of the proteins are indicated by different colors.

**Figure 5:**
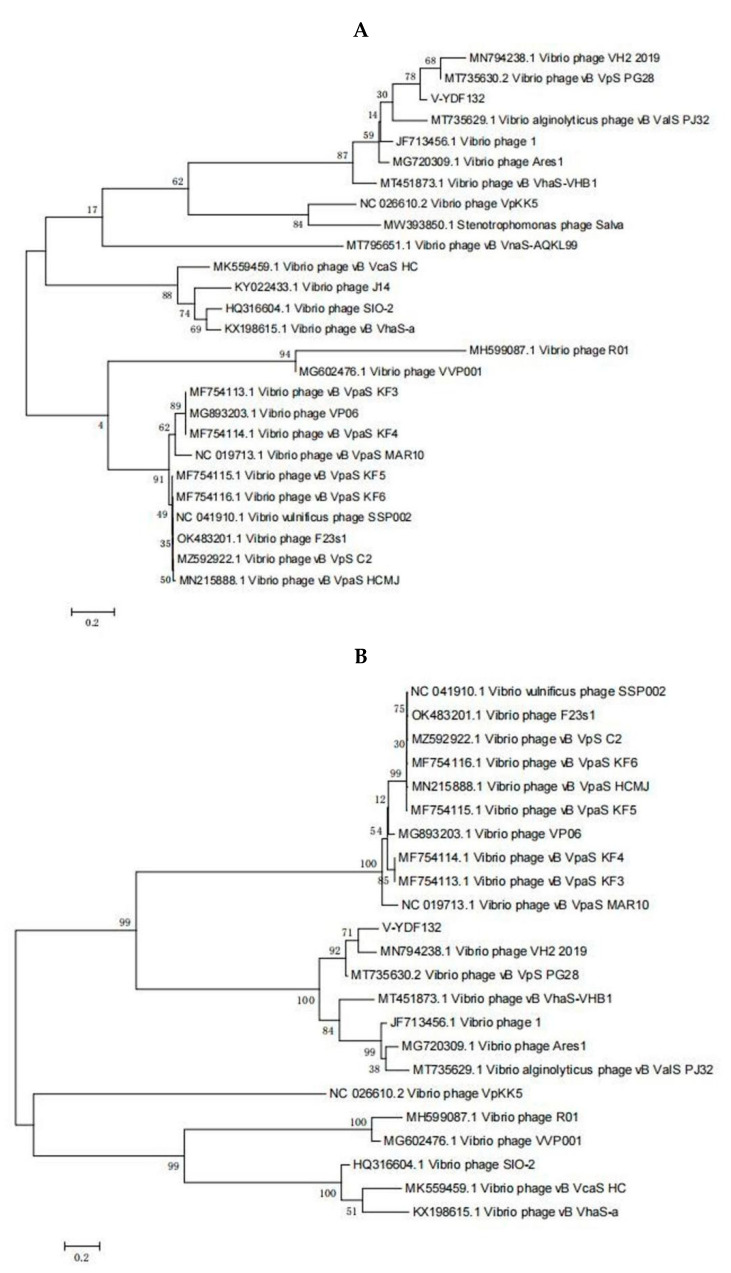
Phylogenetic tree showing V-YDF132 and other related phages of the Siphoviridae family and the Caudovirales order. The amino acid sequences of (**A**) the major capsid protein and (**B**) the terminase large subunit of the related phages were downloaded from the NCBI website.

The genome of V-YDF132 was 84,375 bp in length with a GC content of 46.97%. A total of 115 putative ORFs were predicted in the genome of the phage V-YDF132 (Table 2), with an average gene length of 685 bp and sizes ranging from 186 to 4134 nucleotides. Thirty-eight of the ORFs in the phage genome had predicted functions. The functional genome characteristics, including the position, orientation, and length of each putative gene product, are shown in Table 2. Additionally, 67 ORFs of V-YDF132 are annotated as proteins with unknown functions. No tRNA genes were found in the genome of V-YDF132. The analysis of virulence signature and antibiotic resistance genes demonstrated that no known virulence or resistance genes existed in the genome of V-YDF132. The Bacterial and Archaeal Viruses Subcommittee (BAVS) described the genus as a coalescent group of viruses with high nucleotide sequence similarity (>50%) and using BLASTn, two viruses belonging to the same species differed by less than 5% at the nucleotide level [47]. In the present study, the sequence identity between the phage V-YDF132 and the *Vibrio* phages vB_VpS_PG28 (GenBank accession no. MT735630.2) and VH2_2019 (MN794238.1) was found to be 75.76% and 76.34%, respectively. To further confirm this result, a pairwise comparison of the genome of V-YDF132 and its most closely related phages, *Vibrio* phage vB_VpS_PG28 (MT735630.2) and *Vibrio* phage VH2_2019 (MN794238.1), was performed (Figure 4). According to the previous reports [48,49,50], the ORFs of phage can be divided into six modules, including structure module, DNA metabolism module, packaging module, lysis module, hypothetical module, and other functional modules. Our results demonstrate that V-YDF132 is a newly discovered *Vibrio* phage of the *Siphoviridae* family. As a common practice, phylogenetic trees were edited and visualized to analyze the evolution and recognition of phages. The major capsid proteins and the terminase large subunit are conserved proteins in the phage genome and are often used to assign phages to families by single-gene analysis [51,52]. In the present study, a proteomic comparison of the available amino acid sequences of the major capsid protein and the terminase large subunit was considered to identify distant relatives of the phage by phylogenetic analysis. As shown in Figure 5, both the major capsid protein tree (Figure 5A) and the terminase large subunit tree (Figure 5B) showed that the phage V-YDF132 and the *Vibrio* phages vB_VpS_PG28 (MT735630.2) and VH2_2019 (MN794238.1) could be classified into the same large group. Therefore, it is suggested that these phages may originate from a common distant ancestor. However, the phage V-YDF132 is still distinct from the other phages, suggesting that it represents a new *Vibrio* phage.

### 3.7. Multiple Genome Comparisons and Comparative Genomics

**Figure 6:**
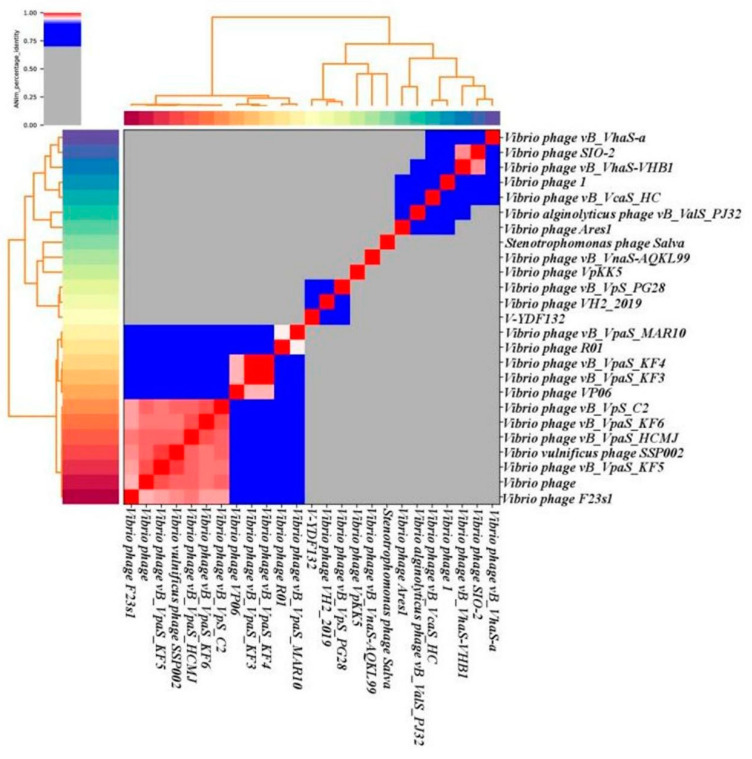
Heatmap of the average nucleotide identity (ANI) values for 25 whole genomes, sequenced from the related phages of the Siphoviridae family in the NCBI database, including phage V-YDF132. Values range from 0 (0%) ANI to 1 (100% ANI): gray represents 0% ANI; clusters of highly similar phages are highlighted in pink and red.

**Figure 7:**
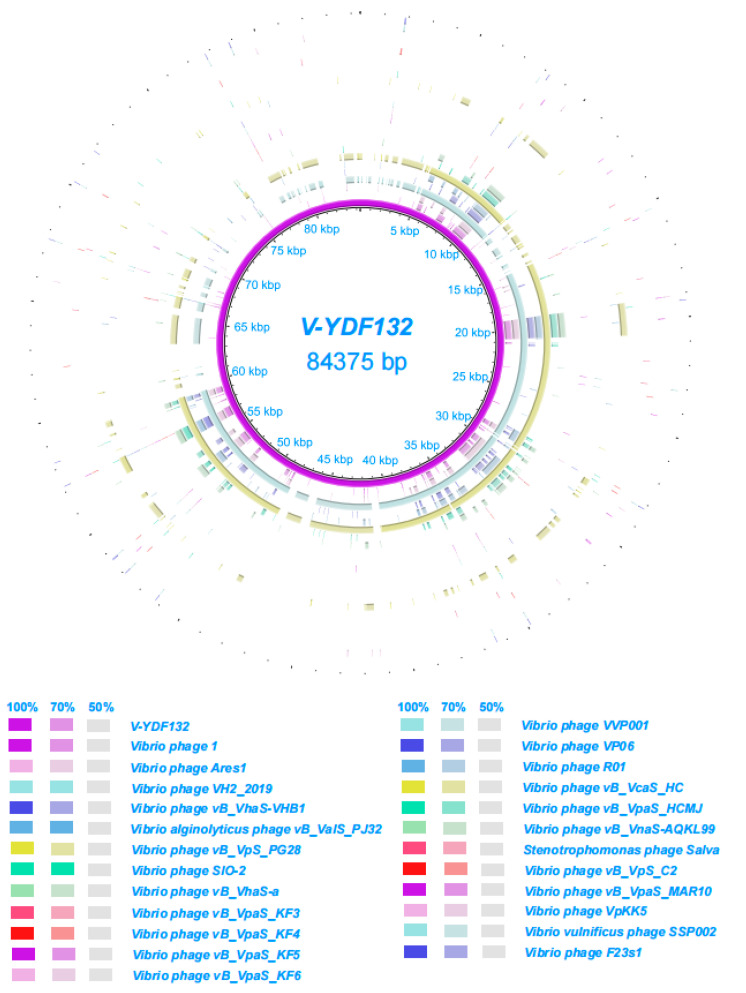
Circular representation depicting the complete genomic comparison of 24 other phages against V-YDF132. The first ring, in purple, represents the genome of V-YDF132. The fragments in different colors outside the purple ring indicate similar regions shared between V-YDF132 and the other 24 phage genomes based on the results from BLAST comparisons.

In Figure 5B, the resulting phylogenetic tree based on the terminase large subunit revealed that V-YDF132 clusters with the Vibrio phages vB_VpS_PG28 (MT735630.2) and VH2_2019 (MN794238.1). To further clarify the relationship between the *Vibrio* phages vB_VpS_PG28(MT735630.2), VH2_2019(MN794238.1), and V-YDF132, an in silico comparison of the genomes of the three phages and the genomes of 22 other phages of the *Siphoviridae* family with distant affinities was performed. Typically, phages within a genus are defined as sharing more than 35% of their genes [53,54]. On analyzing the results of the heatmap (Figure 6) and regarding the ANIm percentage identity data, V-YDF132 and the *Vibrio* phages vB_VpS_PG28 (MT735630.2) and VH2_2019 (MN794238.1) share genes above the threshold value, i.e., 82.88% and 80.97%, which were consistent with those of the phylogenetic analysis. It suggests that V-YDF132 and the Vibrio phages vB_VpS_PG28 (MT735630.2) and VH2_2019 (MN794238.1) belong to the same genus. We also carried out a comparative analysis of the whole-genome sequences of the same phages used in the phylogenetic analyses. Similar to the results of the terminase large subunit analysis, V-YDF132 grouped with the two phages and shared lower similarity with other phages (Figure 7). In conclusion, V-YDF132 is in the same genus as *Vibrio* phage vB_VpS_PG28 (MT735630.2) and VH2_2019 (MN794238.1).

### 3.8. Analysis of the Phage Virus Proteome

The putative gene functions of the phage V-YDF132 were classified into six categories, namely structure module, DNA metabolism module, packaging module, lysis module, hypothetical module, and other functional modules. Six structural proteins were identified in the phage V-YDF132, including the tail tape measure protein, major tail protein, tail completion protein, neck protein, head completion protein, and major capsid protein. ORF104 encodes a DNA methyltransferase, and methylation marks the phage DNA to protect it from the host nucleases, which degrade the host DNA during the early stages of infection [55]. ORF77, ORF87, and ORF102 encode three genes associated with packaging, including the terminase large subunit, the closure protein, and the portal protein, respectively. ORF77 encodes the terminal large subunit, which has ATPase, nucleic acid endonuclease, and DNA decapacitating enzyme activities and consists primarily of two structural domains, namely ATPase and nucleic acid endonuclease [56,57,58,59,60]. Among them, the structural domain of nucleic acid endonuclease has a metal-binding cluster, which is the site of divalent cation binding and is necessary for the large subunit of the terminator enzyme to exert unspinase and nuclease activities [61]. Different phages have different preferences for divalent cations. Therefore, it is speculated that the ATP-dependent zinc metalloprotease encoded by ORF64 is involved in divalent cation binding, and the divalent cation required by the phage V-YDF132 is likely to be the zinc ion. The portal protein encoded by ORF102 along with the terminase large subunit and genomic DNA constitute the packaging machine, and the three collaborate in the packaging process. The portal protein also plays a vital role in the packaging process, which affects the packaging efficiency and is a central component of the packaging sensor. It determines the size of the genome entering the front capsid while maintaining the packaging positivity and preventing the DNA from escaping from the front capsid [62]. The lysin also has a deacetylase encoded by ORF81, a protein structure with a specific cell wall structural domain, and a catalase structural domain, which is a feature commonly found in Gram-negative bacteria. In general, endolysin is required to cross the cell membrane to reach the cell wall layer to cleave peptidoglycan with the help of perforin, but no gene encoding perforin was found in all ORFs. Interestingly, genes related to DNA metabolic modules were identified in our phage particles (ORF12, ORF70, ORF72, ORF101, ORF103, ORF104, and ORF111). They may play an essential role in the process of early phage infection [63], these proteins will be investigated in our future studies.

## 4. Conclusions

In summary, the present study demonstrates that V-YDF132, isolated from a *V. harveyi* strain as the host, is a newly discovered *Vibrio* phage belonging to the *Siphoviridae* family. As characterized by determining its host range, one-step growth curve, and lytic activity, V-YDF132 exhibits strong lytic activity and has a great potential as a newly discovered antibacterial agent for the biological control of vibriosis in aquaculture. Moreover, V-YDF132 is stable up to 50 °C and in pH ranging from 5 to 11; thus, making it relatively more resistant to different environmental factors (pH and temperature). To gain further understanding of V-YDF132, our future work will focus on functional characterization needed to enable biomedical applications of this phage in various fields.

## Figures and Tables

**Table 1:** The tested bacterial strains.

Strain	Bacterial Species	Infectivity
YF.32	*Vibiro harveyi*	−
YF.41	*Vibiro harveyi*	−
YF.42	*Vibiro harveyi*	+
YF.43	*Vibiro harveyi*	−
YF.62	*Vibiro harveyi*	−
YF.72	*Vibiro harveyi*	−
YDF.21	*Vibiro harveyi*	−
YDF.23	*Vibiro harveyi*	−
YDF.132	*Vibiro harveyi*	+
VH1-1	*Vibiro harveyi*	−
VH1-3	*Vibiro harveyi*	−
FJ.12	*Vibiro harveyi*	−
FSK.111	*Vibiro harveyi*	−
FSK.123	*Vibiro harveyi*	−
PH-1	*Photobacterium damselae*	−
VA-1	*Vibrio alginolyticus*	−
AH-1	*Aeromonas hydrophila*	−

Note: clear lysis zone (+) and no lysis zone (−).

**Table 2:** The detected ORFs of V-YDF132, including the length and the putative product.

ORF	Query_Length	Homolog/Species	E Value	%-Identity
1	831	Hypothetical protein	4 × 10^−122^	57.5
2	318	Hypothetical protein	2 × 10^−19^	51.61
3	1611	Hypothetical protein	6 × 10^−12^	23.48
4	387	Hypothetical protein	7 × 10^−32^	54.69
5	450	Hypothetical protein	2 × 10^−61^	67.59
6	432	Hypothetical protein	4 × 10^−22^	38.81
7	438	-		
8	255	Hypothetical protein	1 × 10^−12^	44.05
9	270	Hypothetical protein	1 × 10^−22^	52.87
10	204	Hypothetical protein	5 × 10^−27^	72.31
11	267	Hypothetical protein	6 × 10^−21^	55.13
12	498	RNase H	4 × 10^−63^	61.29
13	549	-		
14	363	Hypothetical protein	2 × 10^−25^	48.25
15	456	-		
16	369	-		
17	1878	Hypothetical protein	2 × 10^−59^	30.9
18	237	Hypothetical protein	5 × 10^−15^	44.87
19	1938	Hypothetical protein	5 × 10^−64^	28.44
20	354	-		
21	339	-		
22	420	Hypothetical protein	5 × 10^−18^	35.82
23	249	Hypothetical protein	4 × 10^−17^	49.33
24	372	Hypothetical protein	2 × 10^−19^	42.98
25	306	-		
26	186	-		
27	840	Polyamine aminopropyltransferase	8 × 10^−12^	60.84
28	336	-		
29	750	Radical SAM protein	1 × 10^−78^	53.36
30	303	Hypothetical protein	4 × 10^−46^	71
31	279	Hypothetical protein	7 × 10^−41^	68.48
32	486	Hypothetical protein	1 × 10^−18^	36.31
33	219	Hypothetical protein	1 × 10^−15^	52.7
34	534	Hypothetical protein	1 × 10^−81^	67.8
35	675	Coil containing protein	2 × 10^−112^	72.32
36	237	Hypothetical protein	2 × 10^−15^	57.58
37	246	Hypothetical protein	1 × 10^−21^	55.56
38	1953	Hypothetical protein	2 × 10^−22^	25.51
39	330	Hypothetical protein	2 × 10^−13^	39.45
40	315	-		
41	315	Hypothetical protein	3 × 10^−40^	63.46
42	657	Hypothetical protein	1 × 10^−42^	36.24
43	630	XkdF	2 × 10^−112^	82.61
44	1206	Transport and binding protein	1 × 10^−139^	65.67
45	948	Major capsid protein	0	91.17
46	288	Hypothetical protein	6 × 10^−24^	61.43
47	609	Head completion adaptor	8 × 10^−90^	66.17
48	459	Neck protein	3 × 10^−88^	81.58
49	444	Tail-completion protein	1 × 10^−76^	77.62
50	798	Major tail protein	1 × 10^−131^	78.87
51	402	Hypothetical protein	5 × 10^−60^	72.93
52	153	Hypothetical protein	3 × 10^−16^	70
53	4134	Tail tape measure protein	0	75
54	387	Hypothetical protein	1 × 10^−64^	75.78
55	1002	Hypothetical protein	1 × 10^−100^	49.4
56	918	Hypothetical protein	1 × 10^−143^	66.89
57	1353	LamG domain-containing protein	3 × 10^−15^	53.51
58	540	Hypothetical protein	2 × 10^−5^	55.49
59	306	Hypothetical protein	2 × 10^−43^	69
60	228	Hypothetical protein	3 × 10^−24^	64
61	462	Hypothetical protein	5 × 10^−87^	78.34
62	546	TMhelix containing protein	2 × 10^−86^	71.11
63	240	Hypothetical protein	2 × 10^−13^	46.34
64	1188	ATP-dependent zinc metalloprotease	0	74.75
65	639	Hypothetical protein	2 × 10^−26^	33.5
66	1221	Hypothetical protein	0	71.77
67	585	Hypothetical protein	4 × 10^−112^	77.32
68	1431	DnaB-like DNA helicase	0	89.5
69	612	Hypothetical protein	2 × 10^−59^	60.95
70	990	DNA primase protein	0	80.85
71	882	Hypothetical protein	2 × 10^−156^	73.83
72	1422	Putative DNA helicase	0	88.25
73	408	Putative DNA-binding domain protein	3 × 10^−52^	71.11
74	366	Hypothetical protein	2 × 10^−57^	71.55
75	1053	Protein RecA	0	91.4
76	390	Hypothetical protein	1 × 10^−56^	67.94
77	1002	Rubredoxin-type fold protein	0	74.77
78	552	Ribonuclease H-like domain protein	2 × 10^−65^	53.07
79	984	Hypothetical protein	4 × 10^−117^	82.08
80	141	Hypothetical protein	2 × 10^−20^	50
81	678	Endolysin	1 × 10^−145^	86.67
82	738	Hypothetical protein	2 × 10^−124^	74.61
83	852	Hypothetical protein	2 × 10^−114^	78.54
84	618	Hypothetical protein	0	86.22
85	750	Hypothetical protein	1 × 10^−124^	67.47
86	1542	Glycosyltransferase	0	79.53
87	768	Nucleoid occlusion protein	2 × 10^−163^	83.53
88	807	Coil containing protein	2 × 10^−157^	82.71
89	1584	Terminase large subunit	0	91.9
90	243	Hypothetical protein	4 × 10^−35^	72.15
91	228	Hypothetical protein	3 × 10^−35^	76.71
92	552	Hypothetical protein	7 × 10^−96^	71.38
93	360	Coil containing protein	7 × 10^−37^	60.36
94	549	Deoxycytidine triphosphate deaminase	1 × 10^−89^	70.49
95	2064	Pyruvate/phosphate dikinase	0	62.19
96	477	Hypothetical protein	1 × 10^−49^	55.8
97	570	Putative protein-tyrosine phosphatase	2 × 10^−92^	49.1
98	993	Hypothetical protein	2 × 10^−129^	57.27
99	528	Hypothetical protein	2 × 10^−66^	59.75
100	897	Transporter	4 × 10^−118^	57.48
101	2340	DNA polymerase	0	87.29
102	1836	Gene transfer agent portal protein	0	75.33
103	1098	ParB-like nuclease domain protein	0	78.63
104	705	DNA methyltransferase	2 × 10^−135^	79.74
105	252	Hypothetical protein	2 × 10^−18^	48.78
106	393	TMhelix containing protein	2 × 10^−54^	60
107	417	Hypothetical protein	3 × 10^−60^	68.35
108	267	Hypothetical protein	2 × 10^−23^	51.69
109	237	Hypothetical protein	2 × 10^−36^	73.08
110	693	Hypothetical protein	2 × 10^−77^	51.08
111	459	Putative DNA polymerase	6 × 10^−41^	47.92
112	261	Hypothetical protein	2 × 10^−13^	43.59
113	528	Hypothetical protein	3 × 10^−23^	37.76
114	501	Hypothetical protein	1 × 10^−49^	49.38
115	300	Hypothetical protein	1 × 10^−15^	34.74

## Data Availability

The datasets generated for this study are available on request to the corresponding author.
